# Heme, an Essential Nutrient from Dietary Proteins, Critically Impacts Diverse Physiological and Pathological Processes

**DOI:** 10.3390/nu6031080

**Published:** 2014-03-13

**Authors:** Jagmohan Hooda, Ajit Shah, Li Zhang

**Affiliations:** Department of Molecular and Cell Biology, Center for Systems Biology, University of Texas at Dallas, Richardson, TX 79489, USA; E-Mails: jagmohan.hooda@utdallas.edu (J.H.); ajitnshah@icloud.com (A.S.)

**Keywords:** heme intake, iron, red meat, cancer, diabetes, coronary heart disease, hemoproteins, cancer progression

## Abstract

Heme constitutes 95% of functional iron in the human body, as well as two-thirds of the average person’s iron intake in developed countries. Hence, a wide range of epidemiological studies have focused on examining the association of dietary heme intake, mainly from red meat, with the risks of common diseases. High heme intake is associated with increased risk of several cancers, including colorectal cancer, pancreatic cancer and lung cancer. Likewise, the evidence for increased risks of type-2 diabetes and coronary heart disease associated with high heme intake is compelling. Furthermore, recent comparative metabolic and molecular studies of lung cancer cells showed that cancer cells require increased intracellular heme biosynthesis and uptake to meet the increased demand for oxygen-utilizing hemoproteins. Increased levels of hemoproteins in turn lead to intensified oxygen consumption and cellular energy generation, thereby fueling cancer cell progression. Together, both epidemiological and molecular studies support the idea that heme positively impacts cancer progression. However, it is also worth noting that heme deficiency can cause serious diseases in humans, such as anemia, porphyrias, and Alzheimer’s disease. This review attempts to summarize the latest literature in understanding the role of dietary heme intake and heme function in diverse diseases.

## 1. Introduction

Proteins are the building blocks of life. They are also the major source of dietary nutrients. When proteins are digested, amino acids are released to the body for biosynthetic purposes or for generating cellular energy. Besides amino acids, proteins also provide other nutrients, particularly metals. Iron is the most abundant metal in the human body; one adult human body needs 3–4 g of iron. Dietary iron is found in two forms, heme and non-heme iron. Heme iron, which is present mainly in meat, poultry and fish, is well absorbed. Non-heme iron, which accounts for the majority of the iron in plants [1], is less well absorbed. More than 95% of functional iron in the human body is in the form of heme [2]. Hence, heme should be considered an essential nutrient for humans, although historically iron is the primary concern in nutrition studies. Particularly, recent studies have shown that heme is efficiently absorbed by the small intestinal enterocytes [3,4]. In Western countries, heme iron derived from myoglobin and hemoglobin makes up two-thirds of the average person’s total iron stores, although it constitutes only one-third of the ingested iron [5]. Evidently, heme is a bona fide essential dietary nutrient. Further, heme directly impacts many physiological and disease processes in humans. In this review, we present the current knowledge of how heme is absorbed in humans and the diseases associated with disturbed heme homeostasis.

## 2. Dietary Heme Is Efficiently Absorbed in the Small Intestine

**Figure 1:**
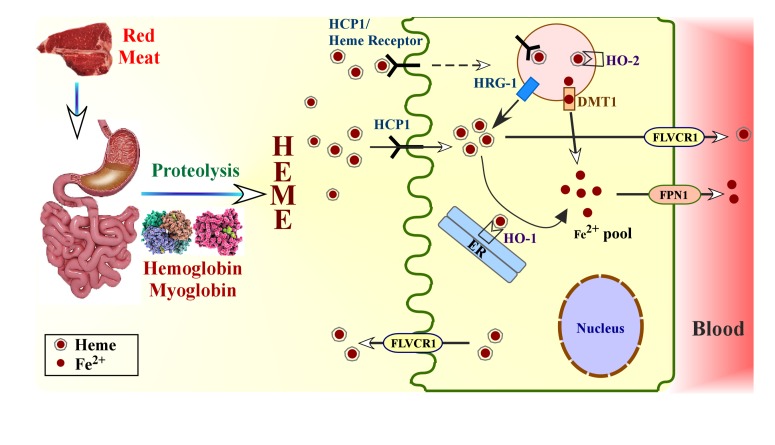
Heme absorption in gut from dietary proteins. Low pH of stomach releases heme-containing proteins hemoglobin and myoglobin from dietary meat. Heme is released by the action of proteases in stomach and intestine. Intake of heme into enterocytes can be facilitated by vesicular transport system when heme binds to heme transporter or heme receptor. Additionally, heme can be directly imported into the enterocytes by HCP1. Heme is transported to the cytoplasm from the vesicles possibly by HRG-1, and is then metabolized by HO-1 present on endoplasmic reticulum. Iron is released subsequently. Alternatively, heme inside the vesicles can be metabolized by the action of HO-2 present on vesicle membrane, and the released iron (Fe^2+^) is transported into the cytoplasm by metal transporter DMT1 to join the common pool of iron in the cytoplasm. Elemental iron is released into the blood stream by the enterocytes via ferroportin present on the basolateral membrane. A fraction of intact heme can be released directly into the blood stream via heme transporter FLVCR1. FLVCR1 exports cytoplasmic heme, and it can export heme into the lumen during increased cellular heme content to protect from heme toxicity. HCP1, heme carrier protein 1; HRG-1, heme responsive gene-1; FLVCR1, cell surface receptor for feline leukemia virus, subgroup C, cellular receptor 1; HO-1/2, heme oxygenase-1/2; DMT1, divalent metal transporter 1; FPN1, ferroportin-1; ER, endoplasmic reticulum.

Heme is found in highest abundance in meat in the form of hemoglobin and myoglobin. Heme is released from these proteins because of the low pH in the stomach and the action of proteolytic enzymes in the stomach and small intestine [5,6] (Figure 1). Concentrated heme produced from hemoglobin hydrolysis in the stomach is poorly absorbed, because pure heme is poorly soluble at the low gastric pH, but the availability of heme is unaffected by gastric secretion [5,6]. There is some evidence that neutralization of gastric contents by pancreatic juice also leads to polymerization of heme, which reduces its availability unless other protein degradation products are present to inhibit polymer formation. The interaction of heme with peptides produced from proteolytic digestion of globin prevents the formation of insoluble heme polymers. Heme solubility is increased significantly by the presence of protein, which is important, given the fact that heme-rich diet have high protein content [4,5,6,7,8,9]. Hence, the peptides and amino acids produced from meat hydrolysis can enhance the absorption of heme and non-heme iron [3,10].

Heme is absorbed by the mucosa as an intact metalloporphyrin [Fe(II)-protoporphyrin-IX] in the lumen by the enterocytes [4,5,11,12] (Figure 1). This may be facilitated by a vesicular transport system, presumably binding first to the brush-border membrane of enterocytes, and then undergoing internalization into the cytoplasm, finally appearing within enclosed vesicles [6]. Internalized heme may be released to the blood via the action of the heme exporter FLVCR1 (Figure 1). FLVCR2 is another putative heme transporter, which might be involved in intracellular heme import [13]. Heme in the blood might be taken up directly by various cells, including liver and erythroid cells, for making hemoproteins. Although heme from dietary hemoglobin has not been demonstrated to be reused directly in animals or humans, heme has been shown to be taken up directly by both intestinal and non-intestinal cells and promote direct cellular responses [14,15,16,17]. Alternatively, heme can be degraded, releasing the iron via the action of heme oxygenase (HO) (Figure 1). Iron then enters the low molecular weight pool of iron in the enterocyte, along with iron absorbed as inorganic non heme-iron (inorganic) iron [4,6,11,18]. In a study tracking the absorption of ^59^Fe-hemoglobin in closed duodenal loops, it is reported that heme degradation is the rate limiting step in heme absorption, as opposed to hemoglobin degradation, heme uptake, or iron transfer to the circulation [5,19]. Subsequently, the elemental iron is released to the bloodstream by the enterocytes via the basolateral transporter ferroportin [4,6,10,11,18]. Absorbed iron in the blood is delivered to the marrow for hemoglobin synthesis, and a small amount is stored mainly in the liver. All of this iron is carried by a single plasma protein known as transferrin. Transferrin, also known as siderophilin, has a molecular weight of about 80 kDa, with two ferric iron binding sites [8].

## 3. The Heme Transporters HCP1, HRG-1 and FLVCR1 Are Important for Maintaining Heme Homeostasis

Research in the past decade has identified several transporters involved in maintaining heme homeostasis [10]. Particularly, the function of three heme transporters have been characterized: proton-coupled folate transporter/heme carrier protein 1 (PCFT/HCP1), heme responsive gene 1 (HRG-1), and cell surface receptor for feline leukemia virus, subgroup C, cellular receptor 1 (FLVCR1) [18,20] (Figure 1). HCP1 has been characterized as a folate/proton symporter and appears to play a key role in intestinal heme and folate absorption. HCP1 is a low affinity heme transporter [5,18,21,22]. It has a higher affinity for folate (Km = 1.67 μM) as compared to heme (Km = 125 μM) [22]. HCP1 mRNA was found to be highly expressed in the duodenal mucosa, which is the main site of intestinal heme absorption. However, virtually no expression is found in the ileum [18]. The highly conserved murine HCP1 is a 50 kDa protein, with 9 predicted transmembrane domains (TM). Xenopus oocytes and HeLa cells expressing HCP1 exhibit 2- to 3-fold increase in heme uptake that is both saturable and temperature dependent [18]. HCP1 is post-translationally regulated in iron deficient mice and transcriptionally under hypoxia. In normal mice, HCP1 resides in the cytoplasm; however, in iron-deficient mice, HCP1 relocalizes from cytoplasm to plasma membrane. Conversely, feeding a high dose of iron to iron-deficient mice redistributed HCP1 from the brush border membrane of the duodenum to the cytoplasm. Levels of HCP1 mRNA are less responsive to iron deficiency but are induced under hypoxia [18,21,22,23]. HCP1 is localized in plasma membrane in non-polarized cells for heme uptake from body fluids, whereas in polarized cells, it is localized in the apical membrane for heme uptake from diet [22]. The post-translational regulation of HCP1 is interesting, because it provides an efficient and fast way to uptake dietary heme before it is lost to gut peristalsis. It also prevents the unnecessary uptake of heme intracellularly when cellular iron content is normal. This mechanism might prevent the accumulation of excess heme and iron, both of which are toxic to cells in excess [10]. It is observed that mRNA expression of HCP1, and by reasoning heme uptake, is regulated by heme [24]. Further investigation is needed to understand the precise mechanism of heme transport via HCP1 [3,5]. Nonetheless, existing studies strongly suggest that HCP1 plays a crucial role in the uptake of heme iron in the intestine, with its expression controlled by the levels of iron and heme in the organism. Recent evidence suggests that HRG-1 is responsible for transporting heme from the endosome to the cytosol [13]. HRG-1 has been reported to be primarily localized on endosome and lysosome related organelles and partially (~10%) on the plasma membrane [13,22,25,26]. It is reported to be expressed on the basolateral, and not apical surface, of polarized Madin-Darby canine kidney cells [22]. HRG-1 is found to be highly expressed in kidney, brain, heart and small intestine [22,26]. The expression of HRG-1 mRNA is tissue dependent, with some studies reporting direct relation between HRG-1 expression and heme concentrations. Transcription factor Bach1 represses anti-oxidant response genes, including HRG-1 and heme oxygenase genes. When intracellular heme concentrations increase, heme binds to Bach1 via HRMs (heme regulatory or responsive motifs) and Bach1 mediated repression is released, Bach1 then dimerizes with the activator protein Maf to stimulate the expression of HRG-1, heme oxygenase and other Bach1 repressed genes [13,27,28,29]. A recent study showed that HRG-1 is the phagolysosomal heme transporter for microphage heme-iron recycling [30]. It mediates heme transport from the phagolysosome during erythrophagocytosis and may play a similar role in the intestine.

FLVCR1, a member of the major facilitator superfamily of transporter proteins, was identified as the cell surface receptor for feline leukemia virus, subgroup C. FLVCR1 is highly expressed in tissues that either transport heme (*i.e.*, intestinal or hepatic cells) or synthesize high levels of heme (erythroid cells). FLVCR1 exports cytoplasmic heme. Heme export by FLVCR1 is time and temperature-dependent; interference with FLVCR1 function blocks heme export and increases cellular heme content [10,20]. FLVCR1 expression is induced during early erythroid differentiation. It functions as a heme exporter to protect the cells from excess heme build up during the CFU-E stage of erythropoiesis. Even under heme deficient conditions, FLVCR1 does not reverse function to increase heme uptake.

## 4. Heme Is Degraded, and Iron Is Recycled by the Action of Heme Oxygenase (HO) in Mammals

HO is localized to the endoplasmic reticulum (ER) and requires NADPH-cytochrome P-450 reductase for its catalytic turnover [22,31,32,33]. It is a rate-limiting enzyme in the catabolism of heme and plays a key role in regulating the intracellular heme levels [34,35]. Three isoforms of HO have been identified so far: HO-1, HO-2 and HO-3. HO-1 is a 32 kDa protein, which can be induced by heme and heavy metals, hyperoxia, hypoxia, UV light, hydrogen peroxide, lipopolysaccharide, hyperthermia or endotoxin [34,35]. HO-1 is transcriptionally regulated, while the 36 kDa protein HO-2 is constitutively synthesized [36]. HO-3 is reported to be derived from a processed pseudogene [22,34,36,37]. HO-1 is expressed in relatively low amounts in all tissues, while HO-2 is constitutive and mainly expressed in brain and testis. HO-1 is constitutively expressed in colonic, gastric and intestinal mucosa [34]. Yanatori *et al*. reported that changes in heme concentrations do not affect the localization of HO-1 in HEp-2 cells [22]. HO expression is highest in duodenum, in which heme absorption is highest, as well as the site for highest expression of HCP1. HO activity increases during iron deficiency [5]. Thus, HO plays important roles in maintaining heme and iron homeostasis.

Besides iron, degradation of heme by HO results in the production of carbon monoxide (CO) and biliverdin IX-α (BV) [34,38]. CO acts as a physiological regulator of cGMP and may function as a neuromodulator [39]. Water soluble biliverdin is released as a result of heme degradation and is further reduced to the orange bile pigment, bilirubin IX-α (BR), by biliverdin reductase (BVR). Bilirubin is then released into the gastrointestinal tract (GI) [10,38,40]. Bilirubin is a lipophilic and water insoluble compound, which is responsible for the yellow color associated with bruises, urine, brown color of feces and yellow discoloration in jaundiced patients [39]. Bilirubin scavenges ROS and is considered to be a potent antioxidant and antinitrosative [34,41]. High baseline serum levels of bilirubin are found to be associated with lower incidences of retinal damage in newborns, reduced risk of ischemia heart disease and reduced rates of cancer-related mortality [40,41]. Bilirubin has antioxidant properties, but unconjugated bilirubin becomes neurotoxic if produced in excess, such as in hemolytic anemia or sepsis. Unconjugated billirubin can result in disruption of cell membrane, a reduction of mitochondrial potential and activation of the apoptotic cascade [42]. Thus, HO activity is responsive to many stimuli, in addition to those related to iron and heme.

## 5. High Heme Intake Is Associated with Increased Risk of Cancer

Dietary differences in the world likely contribute to global variations in cancer cases [43]. Meat is an important source of proteins and provides essential amino acids. It is one of the largest dietary sources of heme [44,45]. Epidemiological and experimental studies have suggested that the high heme content in red meat is associated with several diseases, including heart diseases, diabetes and cancer [45]. Red meat (beef, lamb and pork) has 10-fold high heme content as compared to white meat (chicken) [46]. Studies have shown that an increased risk of several types of cancer is associated with diets high in red meat. On the contrary, consumption of substantial amounts of green vegetables is associated with decreased risk of colon cancer, likely because vegetables contain low levels of heme iron [44,47,48]. Below we provide an overview of recent epidemiological data showing the association of increased risk of cancer with high heme iron intake.

### 5.1. High Heme Intake in Diet Increases the Risk of Colon Cancer

A number of studies have demonstrated a positive association between high intake of red meat and colorectal cancer (CRC). However, the association between red meat intake and other cancer types such as gastrointestinal, lung cancer, pancreatic, breast and esophageal are understudied and less consistent [43,45,49]. Table 1 provides a summary of recent epidemiological studies investigating the association of heme iron intake with an array of diseases, including cancer. Colorectal cancer is the third leading cause of death around the world and accounts for more than 1 million cases and 600,000 deaths each year [44,50]. CRC is most commonly associated with dietary preferences high in red meat, suggesting that the risk of CRC can be reduced by controlling dietary intake [43,51].

A meta-analysis performed in 2006 by Larsson and coworkers showed an elevated overall relative risk (RR) of 1.28 of colorectal cancer (95% confidence interval (CI) = 1.15–1.42) for red meat in the highest versus lowest category of intake. They estimated the RR of 1.28 (95% CI = 1.18–1.39) for an increase of 120 g/day of red meat [52]. Another study suggested an increased risk of colon cancer in men with a diet high in heme and decreased intake of chlorophyll [53]. In 2011 a meta-analysis of prospective cohort studies of colon cancer reported heme intake of 566,607 individuals and 4734 cases of colon cancer [44]. The study compared the RR of subjects with highest category of heme intake with those in lowest category and determined an RR of colon cancer to be 1.18 (95% CI = 1.06–1.32). In their analysis of experimental studies in rats with chemically induced colon cancer, they showed that dietary hemoglobin and red meat promote a putative cancer lesion, an aberrant cryptic foci [44]. Another meta-analysis study showed a significant association between high intake of red and processed meat with increased risk of colorectal, colon and rectal cancers [54]. In this study, the overall RR for colorectal cancer for highest versus lowest intake of fresh red meat for every 100 g/day increase is 1.17 (95% CI = 1.05–1.31). The study showed similar results for colon cancer, but no significant association is found for rectal cancer [54].

In 2013, a meta-analysis further suggested a positive-dose response association of heme intake and the risk for colorectal cancer [50]. In the analysis of eight studies, the authors found a 14% higher risk for CRC in subjects with high intake of heme, compared to the subjects with lowest intake of heme [50]. The observed overall RR for CRC in their study was 1.14 (95% CI = 1.04–1.24) for heme intake [50]. Gene expression profiling of the colon mucosa in mice showed that heme is involved in the downregulation of the inhibitors of proliferation, Wnt inhibitory factor 1, Indian Hedgehog, bone morphogenetic protein 2 and Interleukin-15. The expression of amphiregulin, epiregulin and cyclo-oxygenase-2 mRNA is upregulated by heme in surface cells *vs*. crypt cells. These results suggest that heme inhibits the surface to crypt signaling of feedback inhibitors of proliferation and induces colonic hyperproliferation and hyperplasia, which increases the risk of colon cancer [48].

Several potential mechanisms have been suggested to explain the association between high intake of red meat and the risk of colorectal cancer. Heme is more bioavailable and readily absorbed as compared to non-heme. However, detrimental effects are associated with heme specifically, which includes cytotoxicity and the increased formation of endogenous *N*-nitroso compounds (NOCs), which may increase the overall mutation rate in the DNA of colonic tissue [55,56,57]. Heme in red meat may catalyze the production of endogenous NOCs within the colon and thereby catalyze the formation of cytotoxic and genotoxic aldehydes by lipoperoxidation [43,44,51,56]. It has been shown that under anaerobic conditions, hemoproteins, hemoglobin and myoglobin in meat can react with nitric oxide to form nitrosating agents. In addition, hemes are known to be nitrosating agents and can be easily nitrosated under certain conditions, which is facilitated by the anaerobic and reductive environment of the small intestine by maintaining heme in its ferrous state [56]. Nitrosyl heme and nitroso thiols contribute significantly in the endogenous production of NOCs. Amines and amides produced by bacterial decarboxylation can be *N*-nitrosated in the presence of these nitrosating agents to produce NOCs. A majority of the NOCs investigated are shown to be carcinogens [51,56,58].

### 5.2. Risk of Gastrointestinal and Pancreatic Cancer Is Associated with High Heme Intake

With the availability of large amounts of epidemiological data for colorectal cancer, the positive relation between colorectal cancer and high intake of red meat (high heme content) is convincing; however, limited data is available for other gastrointestinal malignancies. A 2011 study showed a positive association between red meat intake and squamous cell carcinoma of the esophagus [45]. They observed a hazard ratios (HR) of 1.47 (95% CI = 0.99–2.20, *P* for trend = 0.063) for highest versus lowest quintile of heme intake, suggesting a positive association between esophagus adenocarcinoma and heme intake [45]. Jakszyn and coworkers observed a statistical significant association between heme intake and gastric cancer (GC) with HR of 1.13 (95% CI = 1.01–1.26 for a doubling of intake), which was adjusted by age, BMI, sex, tobacco smoking, education level, and energy intake [59]. Their study included 481,419 individuals and 444 cases of GC [59]. Ward and coworkers has also suggested a positive association between the consumption of heme from red meat and increased risk for esophageal and stomach cancer [60].

Pancreatic cancer is one of the most deadly cancers. Hence, previous studies have focused on the association between risk of pancreatic cancer and diet. A meta-analysis performed in 2012 found that there is an increased risk of pancreatic cancer in men, positively associated with consumption of red meat [61]. In that study, RR for risk of pancreatic cancer in men is 1.29 (95% CI = 1.08–1.53), suggesting a significant association of red meat intake and risk of pancreatic cancer. However, the authors did not observe a significant association in women (RR = 0.93, 95% CI = 0.74–1.16) [61].

**Table 1:** Summary of epidemiological studies investigating the association between dietary intake of heme iron and/or red meat with various diseases.

Disease	Diet Intake	HR/OR/RR (95% CI) Highest *vs*. Lowest	Reported Association	Number of Pzarticipants	Age (Years)	Years of Follow Up	Diet Assessment Method	Reference
Colorectal cancer	Red Meat	HR = 1.24 (1.12–1.36)	+	567,169	50–71	8.2	124-item FFQ	[40]
Colon cancer	High heme and low chlorophyll	RR = 1.58 (0.99–2.54)	+	58,279 Men	55–69	9.3	150-item semi quantitative FFQ	[50]
Colorectal cancer with KRAS mutation	Heme Iron	HR = 1.71 (1.15–2.57)	+	4026	55–69	7.3	150-item FFQ	[54]
Esophageal squamous cell carcinoma	Red Meat	HR = 1.79 (1.07–3.01)	+	494,979	50–71	10	124-item FFQ	[42]
	Heme Iron	HR = 1.47 (0.99–2.20)						
Esophageal cancer	Red Meat	HR = 1.51 (1.09–2.08)	+	567,169	50–71	8.2	124-item FFQ	[40]
	Heme Iron	OR = 3.04 (1.20–7.72)	+	124 esophageal, 154 stomach cancer and 449 controls	≥21		100-item Short health habit and history questionnaire	[57]
Gastric cancer	Heme Iron	HR = 1.13 (1.01–1.26)	+	481,419	35–70	8.7	Validated country specific questionnaires	[56]
Stomach cancer	Heme Iron	OR = 1.99 (1.00–3.95)	+	124 esophageal, 154 stomach cancer and 449 controls	≥21		100-item Short health habbit and history questionnaire	[57]
Liver cancer	Red Meat	HR = 1.61 (1.12–2.31)	+	567,169	50–71	8.2	124-item FFQ	[40]
Pancreatic cancer	Red Meat	HR_Men_ = 1.43 (1.11–1.83)	+	567,169	50–71	8.2	124-item FFQ	[40]
Endometrial cancer	Red Meat	HR = 0.75 (0.62–0.91)	inverse association	567,169	50–71	8.2	124-item FFQ	[40]
	Heme Iron	RR = 1.24 (1.01–1.53)	moderate	60,895	Women born between 1914 and 1948	21	67-item FFQ in 1987 and 96-item FFQ in 1997	[62]
Lung cancer	Red Meat	HR_Men_ = 1.11 (0.79–1.56)	No Association	99,579	55–74	8	124-item FFQ	[46]
		HR_Women_ = 1.30 (0.87–1.95)						
	Red Meat	HR = 1.2 (1.10–1.31)	+	567,169		8.2	124-item FFQ	[40]
	Red Meat	HR_Men_ = 1.22 (1.09–1.38)	+	278,380 men and 189,596 women	50–71	8	124-item FFQ	[63]
		HR_Women_ = 1.13 (0.97–1.32)						
	Heme Iron	HR_Men_ = 1.25 (1.07–1.45)						
		HR_Women _= 1.18 (0.99–1.42)						
Type 2 Diabetes	Red Meat	RR = 1.44 (0.92–2.24)	moderate, non-significant	91,246 U.S women	26–46	8	133-item semiquantitative FFQ	[64]
	Red Meat	RR = 1.63 (1.26–2.10)	+	38,394 Men	40–75	12	131-item semiquantitative FFQ	[65]
	Heme Iron	RR = 1.28 (1.04–1.58)	+	35,698 postmenopausal women	55–69	11	127-item FFQ	[66]
	Heme Iron	RR = 1.28 (1.14–1.45)	+	85,031 women	34–59	20	131-item expanded FFQ	[67]
Gestational Diabetes Mellitus	Heme Iron	RR = 1.51 (0.99–2.36)	+	3158 pregnant women	≥18		121-item FFQ	[68]
Myocardial Infarction	Heme Iron	RR = 1.86 (1.14–3.09)	+	4802	≥55	3–7	170-item semiquantitative FFQ	[69]

HR, hazard ratio; OR, odds ratio; RR, relative risk; +, positive association; FFQ, food frequency questionnaire.

### 5.3. High Heme Intake Increases the Risk of Endometrial Cancer in Women

Endometrial cancer accounts for 10–20 per 10^5^ people a year in western countries [62]. An association between heme intake from red meat and the risk of endometrial cancer in women was suggested, but the studies were very limited. Kabat and coworkers [70] (2008) used data from a large cohort study of Canadian women and assessed the risk of endometrial cancer associated with dietary intake of heme. The authors found no association between heme intake and the risk of endometrial cancer [70]. However, a case control study by Kallianpur and coworkers (2010) observed an increased risk of endometrial cancer of ~2-fold with higher heme intake, predominantly after menopause and in women with BMI ≥ 25 kg/m^2^ [71]. Another recent study by Genkinger and coworkers [72] (2012) also suggested a moderately positive association between risk of endometrial cancer and heme intake. The comparison between highest *vs.* lowest quartile in their study showed a 20%–30% of higher risk associated with higher intakes of heme, with a RR of 1.24 (95% CI = 1.01, 1.53 for ≥1.63 compared with <0.69 mg/day) [72]. A few mechanisms have been proposed to explain how heme intake may lead to onset of endometrial cancer: (1) Heme can lead to a higher per-oxidant load and lead to higher oxidative stress and DNA damage; (2) Heme intake is also reported to be associated with increased risk of obesity, diabetes and the markers of obesity and diabetes, which are suspected associated factors for the risk of endometrial cancer [63,72,73,74].

### 5.4. Epidemiological and Molecular Studies Reveal the Link between High Heme Intake and Lung Cancer

Several case control and cohort studies have been reported for an association between high meat (or heme intake) and risk for lung cancer, but these limited studies show inconsistent findings [49]. A study in 2009 reported a strong positive association between intake of heme from meat and lung cancer in men as compared to women [75]. It was found that for association between heme intake and risk for lung carcinoma, hazard ratios (HRs) in comparison with quintiles 5 with 1 (Q5 *vs* Q1) is 1.25 (95% CI = 1.07, 1.45) in men and 1.18 (95% CI = 0.99, 1.42) in women. The study showed an even higher risk of lung cancer in men with high intake of bioavailable heme and lower intakes of antioxidants [75]. On the contrary, Tasevska and coworkers in 2011 reported no significant association between consumption of diet high in red meat and lung cancer [49]. In their study population, they did not observe any association between intake of heme and risk for lung cancer.

**Figure 2:**
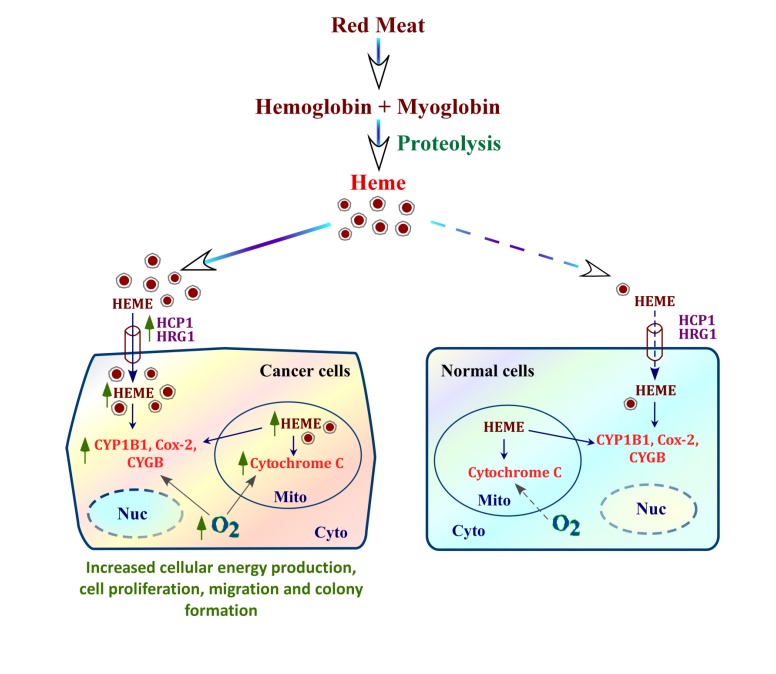
A cartoon illustrating a putative mechanism by which heme fuels cancer cell progression. Heme from blood can be taken up by cells via heme transporters HCP1 and HRG-1. Cancer cells have intensified internal heme synthesis as well as increased heme uptake via heme transporters, whose expression is dramatically elevated in cancer cells, compared to normal cells. As a result, the levels of an array of hemoproteins involving oxygen transport and utilization, such as cytoglobin and cytochrome c, are strongly enhanced. Enhanced levels of hemoproteins lead to intensified oxygen consumption and cellular energy generation, which in turn fuel cancer cell proliferation and migration. HCP1, heme carrier protein 1; HRG-1, heme responsive gene-1; CYP1B1, Cytochrome P450, Family 1, Subfamily B, Polypeptide 1; Cox-2, cyclooxygenase-2; CYGB, cytoglobin; Nuc, nucleus; Mito, mitochondria; Cyto, cytoplasm.

To associate a link between intake of fresh red meat and heme, Lam and coworkers performed a mechanistic study in meat-related lung carcinogenesis by using whole genome expression [65]. They measured the genome-wide expression in tumors and non-involved fresh frozen lung tissues of 64 adenocarcinoma patients. Out of 232 annotated genes in tumor tissue, they found that ~28% (63 genes) were involved in heme transport, absorption (e.g., HFE), binding (e.g., CYP4A11, HPX and NENF), biosynthesis (e.g., ALAS2) and heme/iron mediated wnt signaling pathway (e.g., WNTs, LEF1, PTPRT, TNF) [65]. These results are entirely consistent with recent data gained from molecular studies of lung cancer cells [73]. Notably, a recent molecular study comparing non-small cell lung cancer (NSCLC) cells with nontumorigenic lung epithelial cells suggested a novel, key mechanism underlying heme function in cancer progression [73]. This mechanism can explain the result from epidemiological studies indicating a positive role of heme in promoting cancer. In that study, using a matched pair of cell lines representing normal nonmalignant HBEC30KT and non-small-cell lung cancer (NSCLC) HCC4017 cells developed from the same patient, Hooda and colleagues identified metabolic changes linked with the transformation of normal to cancer cells [73]. They found that oxygen consumption and heme synthesis are intensified significantly in lung cancer cells, compared to the normal cells. Furthermore, the levels of heme uptake proteins and oxygen-utilizing hemoproteins are dramatically increased in cancer cells and xenograft tumors. Inhibition of heme synthesis or mitochondrial function preferentially suppresses cancer cell proliferation, colony formation and cell migration (Figure 2). These results demonstrated that heme availability is significantly increased in cancer cells and tumors, which leads to elevated production of hemoproteins, resulting in intensified oxygen consumption and cellular energy production for fueling cancer cell progression. This provides a unifying mechanism for heme function in promoting cancer progression [73].

In Figure 2, we suggest that dietary heme may be directly reused by cells. This deviates from the traditionally accepted view that dietary heme is degraded in the liver, and iron is released. However, existing data do not counter and are consistent with the idea that heme in the blood can be taken up directly and used by cells. For example, diverse cells, including K562, Caco-2, HepG2 and neuronal cells, are known to directly uptake heme [14,15,16,17]. Thus, under conditions under which heme is needed, as is the case when cancer cells try to proliferate and invade, dietary heme may be released into the blood and taken up by cancer cells to make hemoproteins directly. The highly elevated expression of heme transporters in the cancer cells would drive heme uptake by cancer cells.

## 6. High Heme Intake Is also Associated with an Increased Risk of Type-2 Diabetes and Coronary Heart Disease

### 6.1. High Heme Intake Correlates with Increased Risk of Type-2 Diabetes

Previous epidemiological studies have suggested an association between high heme iron intake and diabetes, as well as coronary heart disease. Diabetes mellitus, also referred to as diabetes, is a growing problem in the modern society characterized by impaired carbohydrate, protein and lipid metabolism caused by insulin resistance and an insufficient amount of insulin secreted. In 2000, the WHO reported that around 47 million people were suffering from diabetes. In 2004, an estimated 3.4 million people had died because of high blood sugar level, with a similar number of deaths reported in 2010. WHO projects that diabetes will be the 7th leading cause of death by 2030 with type-2 diabetes (T2D), accounting for almost 90% of the diagnosed cases [64].

Several cross-sectional and cohort studies have demonstrated positive association between heme intake and T2D [66,67,68,76] (see also Table 1). These studies have been performed in males and females of different ages, and pregnant women. In 1983, Loma Linda University’s Adventist Health Study showed evidence of a positive association between heme from meat and T2D [71]. Since then, several studies have been carried out in different countries to establish this association with different subjects groups [66,67,68,76]. In a large cohort study conducted by Jiang *et al*., authors followed 422,846 persons/year for a period of 12 years from 1986 to 1998 with 1168 reported cases of T2D, to determine possible elevated risk of T2D in men consuming red meat as source of heme intake [66]. They found that men on red meat diet show an increased risk of T2D. However, when chicken, fish, or eggs are the primary source of heme, the association between heme and T2D disappears [66]. In 2004, Lee *et al*. examined the relationship between heme intake and T2D in women [77]. This study was carried out over an 11-year follow-up period [77]. The authors also reported a positive association between heme intake and T2D in women with RR 1.0, 1.07, 1.12, 1.14 and 1.28 across quintiles of heme intake. Interestingly, the association appeared to be stronger among women consuming alcohol, with the risk of T2D increasing with higher alcohol consumption. Particularly, subjects consuming 15 g/day of alcohol have RR across quintiles of heme ranging from 1.0, 2.26, 3.22, 1.92, to 4.42 [77], non-heme iron was inversely associated with incidence of T2D. In 2011, Qui *et al*. showed increased heme intake to be associated with gestational diabetes mellitus (GDM) in pregnant women [78]. Along with dietary heme intake, the association was confounded among women who smoked during pregnancy with a significant RR of 2.09 (95% CI 0.42–10.41), while the corresponding RR for non-smokers is 1.48 (95% CI 0.89–2.46) [78].

The positive association found between heme and T2D is consistent with other studies evaluating the relationship between red meat (heme iron intake) and T2D. The positive association between processed red meat and T2D was confirmed by various studies, suggesting that the risk of T2D increases with higher consumption of processed red meat, regardless of gender, age, ethnicity and BMI [66,67,76,78,79]. Frank Hu and coworkers carried out three cohort studies to evaluate association between unprocessed red meat and T2D [68]. Their result for association of unprocessed red meat with T2D is consistent with that of processed meat. The meta-analyses of the data suggested that a 100 g/day increase in intake of unprocessed meat increases the risk of T2D by 19% (95% CI: 4%–37%) [68] (Table 1).

### 6.2. High Dietary Heme Intake Can Increase the Risk of Coronary Heart Disease Significantly

It is worth noting that previous studies have also demonstrated a positive association between heme and CHD. Ascherio *et al*. reported the earliest evidence of this association in 1994 [80]. They found an increased risk of myocardial infarction among men consuming red meat as the main source of heme and iron [80]. A study of 329 Greek men and women also positively associated the increased risk of CHD among men and women, especially those older than 60 year with an increased dietary heme intake [69]. In another study Snowdon *et al*. used meat intake as a measure of dietary heme and found a 60% increased risk of CHD among men who consume meat six times a week compared with men who consume meat less than once a week [81]. Similar studies have been performed in Netherlands, Italy, USA and Japan [82,83,84,85]. These studies, except the one in Japan, show a statistical correlation between heme intake and increased risk of CHD. Higher heme intake significantly associated with elevated risk of developing CHD with an RR of 31% (95% CI 4%–67%). Meta-analysis of these studies show an 27% increased risk of CHD with a 1 mg/day increase in heme intake. The study in Japan performed by Zhang *et al.* did not show the positive association between heme and CHD [85]. The source of heme in Japanese diet is mainly from fish. It is likely that levels of heme in fish are not high enough to cause CHD. Also, fish and shellfish are excellent source of vitamin D and *n*-3 fatty acids, which protect against CHD [86,87]. Therefore, the dietary profile may have obscured the association between heme and CHD in this study.

### 6.3. Multiple Mechanisms May Underlie the Association of Heme Intake with T2D and CHD

Although the exact mechanism for association of increased dietary heme with T2D and CHD is uncertain, several mechanisms have been proposed. In western countries, heme in the diet is mainly derived from red meat. The feedback mechanism for iron absorption functions better for non-heme iron as compared to that for heme. At any serum ferritin level, the percentage of heme absorbed is higher compared to non-heme iron. As a result, heme continues to be absorbed by the body even in events of excess serum ferritin [88]. Excess of heme intake also leads to iron overload in the body. Iron is a strong pro-oxidant and catalyzes production of reactive oxygen species in various cellular reactions [89]. Excess of iron causes damage to various tissues, especially pancreatic beta cells through increasing oxidative stress by the reactive oxygen species generated, resulting in the damage of insulin production and excretion [90].

Additionally, excessive heme intake leads to deposition of iron deposition in pancreatic beta cells and other tissues, which induces insulin resistance [91,92]. The highly active iron damages the DNA and cell integrity and interferes with glucose uptake by various tissues. An excess of iron was shown to diminish utilization of glucose by muscle tissues [92,93]. This results in shift from glucose utilization to fatty acid production [92,93]. The highly reactive hydroxyl free radicals generated by iron promote oxidation of low-density lipoprotein cholesterol [88,94]. Two studies have indicated that macronutrients like sodium and nitrites present in the meat along with heme increase the risk of type-2 diabetes [95,96]. A recent study in Finland suggested that sodium in the processed red meat contributes to T2D [96]. In addition, the nitrites used in the preservation of red meat enter the body as nitrosamines [95]. These nitrosamines have been shown to be toxic to pancreatic beta cells and increase the risk of type 2-diabetes. Therefore, from a health point of view, a decrease in the red meat consumption, particularly processed red meat, and substitute with other source of heme, such as chicken, eggs, fish, nuts, dairy products and whole grains, should be considered to decrease potential T2D and CHD risk.

## 7. Heme Deficiency Can Cause Serious Health Problems in Humans

While high dietary intake of heme may have adverse effects on health, heme deficiency can also cause serious health problems. In the human body, roughly 80% of heme is present in red blood cells, 15% is synthesized and present in liver, and the rest is distributed in other tissues [97,98,99]. Previously, Tatyana and coworkers have summarized the following newly identified roles of heme: (1) Role in gene expression regulation in yeast Hap1, mammalian Bach1 and bacterial Irr transcription factor proteins; (2) Role in controlling the ion channels (such as, high conductance voltage and Ca^2+^ activated potassium channels); (3) Role in binding to different nuclear receptor proteins; (4) Role in the regulation of circadian clock (day-night cycle); (5) Role in erythrocytes [98].

### 7.1. Defects in Heme Biosynthesis Can Cause Anemia and Porphyrias in Humans

Heme biosynthesis involves eight enzymes, and a defect in any one of them is associated with diseases. Such a defect can be caused by genetic mutations or environmental factors that suppress heme synthesis, such as the presence of lead or lack of iron. Defective heme synthesis can cause acute porphyrias, which are associated with neurological problems in peripheral nervous system (e.g., motor weakness and sensory changes, such as abdominal pain, paresthesia and loss of sensation) and CNS (e.g., insomnia, depression, anxiety, hallucination, seizures and paranoia) [100]. Sideroblastic anemia is a rare X-linked disease, which is caused by the defect in the erythroid-specific enzyme 5-aminolevulinic acid synthase-2 (ALAS2). Defects in any of the other seven enzymes are associated with porphyria [100]. Porphyrias are divided into erythropoietic porphyrias and acute hepatic porphyrias [100]. Neuronal problems are associated with the following four known types of hepatic porphyrias: 5-aminolevulinate dehydratase deficient porphyria (ADP), acute intermittent porphyria (AIP), hereditary coproporphyria (HCP), and variegate porphyria (VP) [100]. Three out of four hepatic porphyrias are dominantly inherited, except ADP, which is rare and a dominant recessive disease [101]. Overproduction and accumulation of porphyrins and porphyrin precursors, 5-aminolevulinic acid (ALA) and porphobilinogen (PBG) represents the clinical manifestations of the porphyrias. Besides genetic defects, lack of iron can also cause heme deficiency, as is the case in iron deficiency anemia. Lack of iron can be caused by low dietary iron intake, as in the case of vegetarians, or by blood loss, for example, heavy menstrual bleeding.

### 7.2. Heme Regulates Diverse Neuronal Genes

Heme is involved in the regulation of neuronal specific genes, particularly through the NGF signaling pathway [98,102]. It has been reported that heme deficiency induces apoptosis in NGF-induced neuronal cell lines. Heme deficiency induced pro-apoptotic JNK signaling pathway and inactivated the pro-survival Ras-ERK1/2 signaling pathway [103]. One possible mechanism of heme regulatory action may be through modulation of kinase activity. Work in the Zhang lab showed that heme actively interacts with Jak2 and Src and affects the phosphorylation of key tyrosine residues in Jak2 and Src [104]. In addition, our microarray data showed that heme deficiency in NGF-induced rat neuronal cells altered the activity of several important neuronal specific genes. They include the structural genes encoding neurofilament proteins and synaptic vesicle proteins, regulatory genes encoding signaling components β-arrestin and p38 MAPK, and stress-response genes encoding hsp70 [103].

### 7.3. Altered Heme Metabolism Is Associated with Alzheimer’s Disease

Alzheimer’s disease (AD) is an age-related neurodegenerative disorder, a common cause of age-related dementia. It is characterized by memory loss caused by a decline in synaptic function, the formation of neurofibrillary tangles, and neuronal cell death, which is followed by a further decline in cognitive and physical functions. Amyloid-β (Aβ) peptide aggregation is associated with the pathophysiology of AD and is associated with the loss of iron homeostasis and mitochondrial complex IV [98,105]. Altered heme metabolism is found in the brains of AD patients [98]. Defective heme metabolism is associated with aberrations in the electron transport chain (loss of complex IV), dimerization of APP, free-radical production, markers of oxidative damage and ultimately cell death, which represents key cytopathologies of AD [106]. The expression levels of heme synthetic enzymes ALAS1 and porphobilinogen deaminase are substantially decreased, which may be associated with the heme deficiency seen in AD, suggesting that the heme biosynthesis is altered in AD patients. The expression levels of heme oxygenase (HO) have been reported to be increased in the cerebral cortex and hippocampus in AD patients [35]. There are conflicting views about the effects of HO; it is not clear whether HO is associated with heme deficiency or an increased heme synthesis [98]. Due to increased heme degradation in AD patients, the levels of bilirubin, a product of heme degradation, are increased in the CSF of AD patients [107]. Aβ binds two molecules of heme with a binding constant of K_a1_ = 7.27 × 10^−6^ M^−1^ (*n*_1_ = 1.5) and K_a2_ = 2.89 × 10^–6^ M^−1^ (*n*_2_ = 1.8), forming Aβ-heme complex, which may lead to the functional heme-deficiency in the brains of AD patients [108]. Heme binding with Aβ prevents the formation of Aβ aggregates, although the Aβ-heme complex is a peroxidase and can oxidize several biomolecules. Sequestration of heme by Aβ creates a heme-deficient environment leading to dysfunctional mitochondria and altered metabolic activity in AD brain [106].

### 7.4. Heme Is Important in the Regulation of Circadian Rhythm in Humans

Circadian rhythm is the clock governing many important behaviors and physiological processes in humans, such as sleep/wake cycle, feeding, body temperature, hormone secretion, and metabolism [109]. Molecular and biochemical studies suggested an involvement of heme in the regulation of circadian rhythms. Neuronal PAS domain protein 2 (NPAS2) is a heme-binding protein and plays a role in the regulation of circadian rhythms [110,111]. DNA binding activity of heme-bound NPAS2 can be inhibited by low micromolar concentrations of carbon monoxide, suggesting that the expression of its target genes are regulated by the gas through a heme-based sensor [110,111]. Heme acts as a ligand for the nuclear hormone receptors REV-ERBα and REV-ERBβ. REV-ERBα regulates a number of physiological functions, including circadian rhythms and metabolic gene pathways. It acts as a heme sensor for the coordination of circadian and metabolic pathway [109,112,113]. A study in mice suggests that a defect in heme biosynthesis, especially heme deficiency, can affect the phase and period of circadian clock [114]. Thus, heme acts as a factor modulating the function of mammalian circadian clock genes [114].

## 8. Conclusions

This review offers a comprehensive analysis of the current literature on the health benefits and risks of heme as an essential nutrient. Heme constitutes 95% of the functional iron in the human body and accounts for two-thirds of iron intake for people in Western countries. Therefore, heme ought to be a crucial factor when considering human nutrition and health. This review summarizes both epidemiological and molecular studies regarding heme function in health and diseases. It can serve as a starting point for further discussion of heme function as an essential nutrient and investigation of heme function in the pathogenesis of diverse diseases.
